# Medication Competence, Numeracy, and Health Literacy

**DOI:** 10.3928/24748307-20190625-01

**Published:** 2019-08-08

**Authors:** Kevin M. Gutierrez, Lawrence D. Cohn

## Abstract

Two studies investigated the association between medication literacy and numeracy. Study 1 revealed an association between both variables in a sample of adults. Study 2 replicated the finding in a sample of parents of young children, even after controlling for level of parental health literacy. Both studies employed the Medication Literacy in Spanish and English (MedLitRxSE) assessment tool. Objective and subjective numeracy scores were significantly correlated with MedLitRxSE scores in both studies, suggesting that interventions for reducing medication errors might benefit by simplifying the numerical information presented in medication instructions. **[*HLRP: Health Literacy Research and Practice*. 2019;3(3):e181–e186.]**

Approximately 90 million adults in the United States display limited health literacy, failing to adequately process, understand, and respond to basic health information (Kindig, Panzer, & Nielsen-Bohlman, 2004). These difficulties can adversely affect the proper administration of over-the-counter medications, which are used on a regular basis by more than 40% of U.S. adults (Wolf et al., 2012). Low health literacy can also adversely affect the proper administration of prescription medications, which are estimated to be used on a monthly basis by at least 69% of adults age 45 to 64 years and 90% of adults age 65 years and older (National Center for Health Statistics, 2016). Low health literacy is associated with misunderstanding medication instructions, poor medication self-management, and reduced medication adherence (Estrada, Martin-Hryniewicz, Peek, Collins & Byrd, 2004; Lokker et al., 2009; Osborn et al., 2013; Stilley, Terhorst, Flynn, Fiore, Stimer, 2014; Yin et al., 2009). Thus, many U.S. adults are at risk for a range of poor health outcomes due to limitations in medication competence and general health literacy.

Children are particularly susceptible to the deleterious consequences of medication dosing errors and medication mismanagement. Many dosing errors occur at home, and recent research suggests that at least 45% of parents commit medication dosing errors (Harris et al., 2017). Approximately 46% of a large representative sample of 6,100 U.S parents incorrectly answered at least 1 of 2 medication-related questions included in the National Assessment of Adult Literacy (Yin et al., 2009). Similarly, in a randomized controlled study of 2,110 parents of children age 8 years or younger, 84% of the parents made at least one medication dosing error, and 21% of parents made at least one large error (using twice the prescribed medication dose indicated on the drug label) (Yin et al., 2016).

An essential component of health literacy involves the capacity to understand and use numerical information provided in medication instructions. The capacity to use numerical information in health-related contexts is a form of “health numeracy,” (Cokely, Galesic, Schulz, Ghazal, & Garcia-Retamero, 2012; Peters & Bjalkebring, 2015) formally defined as “the degree to which individuals have the capacity to access, process, interpret, communicate, and act on numerical, quantitative, graphical, biostatistical, and probabilistic health information needed to make effective health decisions”(Goldbeck, Ahlers-Schmidt, Paschal, & Dismuke, 2005, p. 375). The correct use of numerical information in medical contexts has become increasingly important as health care has become increasingly patient-centered, requiring adults to actively monitor their own medication use and the medication use of children or aging parents. Yet, few studies have investigated the relationship between numeracy and medication literacy.

The two studies reported here address this gap in knowledge. Study 1 tested the hypothesis that adult numeracy is significantly associated with medication literacy. Study 2 modified the latter hypothesis in two important ways. First, the study assessed the relationship between numeracy and medication literacy in a sample of adults who were parents of young children. Young children represent the population at greatest risk for deleterious consequences of medication errors, so investigating factors that influence parental medication literacy is critical for developing effective interventions for reducing medication mismanagement. Approximately 4.8 million undergraduate students are parents, representing 26% of all U.S. undergraduates; 76% of these “student-parents” are women (Gault, Reichlin Cruse, Reynolds, & Froehner, 2014). Notably, all parents in our study had completed high school but they had not yet completed college, thus providing insight into factors influencing medication literacy in a large segment of the community. In addition, Study 2 assessed the relationship between numeracy and medication competence after controlling for the influence of general health literacy. Most studies investigating the relationship between numeracy and medication competence have not included general measures of health literacy, precluding an investigation of the degree to which numeracy skills are associated with medication competence beyond the influence of general health literacy. Study 2 addressed the latter gap in knowledge. Both Study 1 and Study 2 employed correlational designs to test their respective hypotheses.

## Study 1

### Participants

Participants were 101 young adults (73% women, 84% Hispanic) recruited from the University of Texas at El Paso, a large urban university located in the southwest along the U.S.-Mexico border. Approximately 42.6% of the participants were freshmen, 24.8% sophomores, 13.9% juniors, 15.8% seniors, and 3% who were unsure of their academic status. The mean age of the sample was 21.96 years (standard deviation [*SD*] = 5.46).

### Measures

Participants completed a 28-item demographic and medical questionnaire assessing age, gender, ethnicity, and native language, an 11-item objective numeracy scale (ONS) (Lipkus, Samsa, & Rimer, 2001), an 8-item subjective numeracy scale (SNS) (Fagerlin et al., 2007), and the 14-item Medication Literacy in Spanish and English (MedLitRxSE) assessment tool (English version) (Nguyen et al., 2015; Sauceda et al., 2012). The MedLitRxSE tool provides respondents with four scenarios involving the administration or use of medication for oneself or for a family member, such as administering acetaminophen to one's niece or administering a diabetes medication to one's mother. Each scenario in the MedLitRxSE is followed by a series of questions regarding the medication or prescription label, including proper dosage, timing, or administration of the medicine. Responses to each question are coded as correct (1) or incorrect (0). Total score can range from 0 to 14. The instrument is available in both English and Spanish; the English version was employed in the present study. The measure displays good internal consistency (Kuder–Richardson [KR]-20 = 0.78 in English version; KR-20 = 0.80 in Spanish version) (Sauceda et al., 2012).

### Procedure

Participants completed tasks in the following order: consent form, demographic and medical questionnaire, medication competence task, subjective numeracy task, and objective numeracy task. Tasks were administered individually to participants in a small room within a research laboratory; the lead author (K. M. G.) and associated research assistants administered the tasks, which required approximately 45 minutes to complete. Participants earned research credit for their participation. Approval to conduct the study was obtained from the University of Texas at El Paso's Institutional Review Board.

### Results and Discussion

Mean ONS and medication literacy scores were 7.58 (*SD* = 2.52) and 11.17 (*SD* = 1.42), respectively. Significant inter-correlations were obtained between medication literacy scores and objective and subjective numeracy scores (**Table 1**).

Hierarchical regression analysis assessed the association between medication competence and numeracy as measured by the ONS (**Table 2**). In the first step, we entered age, English proficiency, family and/or self-diabetes status, current use of prescription medication, administration of over-the-counter (OTC) medication in the past year, and past year use of OTC medication. In the second step, we entered ONS scores. Age and ONS scores each accounted for a significant amount of variance in MedLitRxSE scores (7.8% and 14.7%, respectively; *p* values < .01).

The findings suggest that numeracy is significantly associated with medication competence. Study 2 sought to replicate this finding in a sample of parents with young children. Study 2 also sought to determine if the association between numeracy and medication competence remains significant after controlling for parental health literacy.

## Study 2

### Participants

One hundred and fifteen parents (73% women; 79% Hispanic) of young children were recruited from the same large urban university (University of Texas at El Paso) that served as the recruitment site in Study 1. The mean age of the parents was 27.6 years (*SD* = 7.28). All participants had at least one child younger than age 13 years. The mean number of children in each family was 1.5; the mean age of the youngest child per family unit was 3.9 years (*SD* = 3.5). Flyers announcing the study were posted in several university venues, including the university daycare center, health center, student union, and lecture halls. Participants were paid $20 for their participation or, instead, were awarded research credit when requested.

### Measures

Participants completed several measures, including a demographic and background questionnaire that had several items assessing prior medical and medication experiences (e.g., “How often have you taken prescription medication during the past 30 days?” or “How often have you taken over-the-counter medication during the past 30 days?”). Participants also completed a measure of objective numeracy (ONS) (Lipkus et al., 2001), subjective numeracy (SNS) (Fagerlin et al., 2007), and the 36-item Short Test of Functional Health Literacy in Adults (STOFHLA) (Baker, Williams, Parker, Gazmararian, & Nurss, 1999). Participants also completed the 14-item MedLitRxSE assessment tool (English version) (Sauceda et al., 2012).

### Procedure

Participants completed questionnaires and tasks in the following order: consent form, demographic questionnaire, medication competence task, SNS, ONS, and STOFHLA. The protocol was administered individually to participants in a small room within a research laboratory; the lead author (K. M. G.) and associated research assistants administered the tasks, which required approximately 45 minutes to complete. Approval to conduct the study was obtained from the University of Texas at El Paso's Institutional Review Board.

### Results and Discussion

Mean ONS, medication literacy, and general health literacy scores were 7.4 (*SD* = 2.3), 12.3 (*SD* = 1.4), and 34.9 (*SD* = 0.08), respectively. Intercorrelations among scores on the SNS, ONS, MedLitRxSE, and STOFHLA are presented in **Table 3**. As predicted, significant associations were revealed among ONS, MedLitRxSE, and STOFHLA scores. Hierarchical regression analyses revealed that both health literacy and numeracy accounted for significant variance in medication literacy scores after controlling for age, English proficiency, past month use of prescription medication, past month use of OTC medication, past month administration of prescription medication, and past month administration of OTC medication (**Table 4**). Specifically, STOFHLA scores uniquely accounted for 17.1% of the variance in MedLitRxSE scores, and ONS scores uniquely accounted for an additional 3.9% of the variance in MedLitRxSE scores. Both findings suggest that medication literacy is distinct from numeracy and general health literacy, although medication literacy is uniquely influenced by both variables.

## General Discussion

The current findings support the hypothesis that numeracy skills represent a unique component of medication literacy. Assessing numeracy skills in parents and patients may be an important step toward reducing medication errors and mismanagement. The moderately strong correlations between subjective and objective numeracy scores in both studies suggest that administering a brief subjective numeracy assessment to patients may be an efficient strategy for evaluating a patient or caregiver's numeracy skills. Completing a brief measure of subjective numeracy may be perceived as less threatening, less stressful, and less frustrating than completing a measure of objective numeracy (Fagerlin et al., 2007). Assessing the numeracy skills of parents, older adults, and caregivers may be particularly important for reducing medication mismanagement because young children and older adults are particularly vulnerable to the deleterious effects of over-medication. Several medication-literacy measures have recently been developed, but only one study has investigated the association between numeracy skills and medication competence. A study of 205 clinic patients, age 18 to 80 years, revealed a significant association between scores on the Medication Understanding Questionnaire and scores on the numerical portion of the Wide Range Achievement Test (*r* = 0.24, *p* < .001) (Osborn et al., 2013). The present study extends the latter finding and suggests an association between numeracy and medication competence that is independent of the influence of general health literacy. It is important to note that medication literacy scores and STOFHLA scores were relatively high and displayed little variability. High scores and low variability on these measures could have influenced the relationship that was revealed between numeracy and medication competence. The high scores and limited variance may have attenuated the strength of the observed relationship. Future research would benefit from recruiting participants who display a wider range of medication competence and health literacy.

The current study was not designed to investigate the impact of gender on the relationship between numeracy and medication competence, and future studies would benefit from research designs that rigorously address this issue. Future studies would also benefit by investigating the association between numeracy and medication competence in adults who have never attended college (a population that includes more than 85 million people age 25 years or older) (U.S. Census Bureau, 2014). Adults who are enrolled in college may have higher health literacy and better numeracy skills than adults who have not attended college. Indeed, health literacy scores, as measured by the STOFHLA, were high in the current research. Somewhat surprisingly, however, is that numeracy skills, as measured by the ONS, were low in this college sample. The latter finding is consistent with some past research reporting low numeracy in samples of highly educated people. Finally, the current research focused solely on English-proficient participants, yet more than 25 million adults in 2013 were characterized by limited English proficiency (LEP) (Zong & Batalova, 2015). Investigating the relationship between numeracy and medication competence in adults with LEP will also be critical for advancing proper medication management in the U.S.

## Figures and Tables

**Table 1:** Intercorrelations Among Scores on the SNS, ONS, and the MedLitRxSE Assessment Tool in Study 1

**Measure**	**1**	**2**	**3**
SNS	.78		
ONS	.56^**^	.82	
MedLitRxSE	.25^*^	.44^**^	.78

Note. Numbers on diagonal are Cronbach's alpha coefficients. MedLitRxSE = Medication Literacy Scale in Spanish and English; ONS = objective numeracy scale; SNS = subjective numeracy scale.

* *p* < .05.

** *p* < .01.

**Table 2:** Summary of Hierarchical Regression Analyses for Variables Predicting MedLitRxSE Scores in Study 1

**Variable**	***R^2^***	**B**	***SE* B**	**Beta**	**Unique Variable (%)**

Step 1	.21				
Age^**^		.08	.03	.31	9.1
English proficiency		.05	.03	.16	
Family/self-diabetes status		.19	.3	.06	
Current use of prescription medication		.11	.33	.03	
Administration of OTC medication in past year	.35	.32	.11	-	
Use of OTC medication in past year		.54	.48	.11	

Step 2	.35				
Age^**^		.08	.02	.29	7.8
English proficiency		.04	.03	.15	
Family/self-diabetes status		.13	.27	.04	
Current use of prescription medication		.43	.31	.13	
Administration of OTC medication in past year		.08	.30	.02	
Use of OTC medication in past year		.41	.44	.09	
ONS composite^**^		.25	.06	.41	14.7

Note. B = unstandardized beta; MedLitRxSE = Medication Literacy Scale in Spanish and English; ONS = objective numeracy scale; OTC = over-the-counter; SE = standard error.

** *p* < .01.

**Table 3:** Intercorrelations Among Scores on the SNS, ONS, MedLitRxSE Assessment Tool, and STOFHLA in Study 2

**Measure**	**1**	**2**	**3**	**4**
SNS	.73			
ONS	.32^**^	.68		
MedLitRxSE	.19^*^	.29^**^	-	
STOFHLA	.01	.34^**^	.41^**^	.48

Note. Numbers on diagonal are Cronbach's alpha coefficients. MedLitRxSE = Medication Literacy Scale in English and Spanish; ONS = objective numeracy scale; SNS = subjective numeracy scale; STOFHLA = Short Test of Functional Health Literacy in Adults.

* *p* < .05.

** *p* < .01.

**Table 4:** Summary of Hierarchical Regression Analyses for Variables Predicting MedLitRxSE Scores in Study 2

**Variable**	***R^2^***	**B**	***SE* B**	**Beta**	**Unique Variable (%)**

Step 1	.08				
Age		.03	.02	.15	
English proficiency		−.17	.11	−.17	
Administration of OTC medication in past month		.03	.02	.17	
Administration of Rx medication in past month		−.01	.01	−.10	
Use of OTC medication in past month		.01	.03	.02	
Use of Rx medication in past year?		.01	.02	.05	

Step 2	.33				
Age		.02	.02	.1	
Language proficiency		−.17	.1	−.17	
Administration of OTC medication in past month		.03	.02	.17	
Administration of Rx medication in past month		−.01	.01	−.11	
Use of OTC medication in past month		−.01	.02	−\.06	
Use of Rx medication in past year?		.01	.02	.06	
STOFHLA score^**^		.69	.19	.39	17.1
ONS score^*^		.14	.07	.22	3.9

Note. B = unstandardized beta; MedLitRxSE = Medication Literacy Scale in Spanish and English; ONS = objective numeracy scale; OTC = over-the-counter; Rx = prescription; SE = standard error; STOFHLA = Short Test of Functional Health Literacy in Adults.

* *p* < .05.

** *p* < .01.

## References

[x24748307-20190625-01-bibr1] Baker D. W. Williams M. V. Parker R. M. Gazmararian J. A. Nurss J. (1999). Development of a brief test to measure functional health literacy. Patient Education and Counseling, 38(1), 33–42. 10.1016/S0738-3991(98)00116-5 14528569

[x24748307-20190625-01-bibr2] Cokely E. T. Galesic M. Schulz E. Ghazal S. Garcia-Retamero R. (2012). Measuring risk literacy: The Berlin Numeracy Test. Judgment and Decision Making, 7, 25–47. 10.1037/t45862-000

[x24748307-20190625-01-bibr3] Estrada C. A. Martin-Hryniewicz M. Peek B. T. Collins C. Byrd J. C. (2004). Literacy and numeracy skills and anticoagulation control. American Journal of Medical Science, 328, 88–93. 10.1097/00000441-200408000-00004 15311167

[x24748307-20190625-01-bibr4] Fagerlin A. Zikmund-Fisher B. Ubel P. A. Jankovic A. Derry H. Smith D. M. (2007). Measuring numeracy without a math test: Development of the Subjective Numeracy Scale. Medical Decision Making, 27, 672–680. 10.1177/0272989X07304449 17641137

[x24748307-20190625-01-bibr5] Gault B. Reichlin Cruse L. Reynolds E. Froehner M. (2014). 4.8 million college students are raising children. Retrieved from Institute for Women's Policy Research website: https://iwpr.org/publications/4-8-million-college-students-are-raising-children/

[x24748307-20190625-01-bibr6] Golbeck A. L. Ahlers-Schmidt C. R. Paschal A. M. Dismuke S. E. (2005). A definition and operational framework for health numeracy. American Journal of Preventive Medicine, 29, 375–376. 10.1016/j.amepre.2005.06.012 16242604

[x24748307-20190625-01-bibr7] Harris L. M. Dreyer B. P. Mendelsohn A. L. Bailey S. C. Sanders L. M. Wolf M. Yin H. S. (2017). Liquid medication dosing errors by Hispanic parents: Role of health literacy and English proficiency. Academic Pediatrics, 17, 403–410. 10.1016/j.acap.2016.10.001 28477800 PMC5424611

[x24748307-20190625-01-bibr8] Kindig D. A. Panzer A. M. Nielsen-Bohlman L. (Eds). (2004). Health literacy: A prescription to end confusion. Retrieved from National Academies Press website: https://www.nap.edu/catalog/10883/health-literacy-a-prescription-to-end-confusion 25009856

[x24748307-20190625-01-bibr9] Lipkus I. M. Samsa G. Rimer B. K. (2001). General performance on a numeracy scale among highly educated samples. Medical Decision Making, 21, 37–44. 10.1177/0272989X0102100105 11206945

[x24748307-20190625-01-bibr10] Lokker N. Sanders L. Perrin E. M. Kumar D. Finkle J. Franco V. Rothman R. L. (2009). Parental misinterpretations of over-the-counter pediatric cough and cold medication labels. Pediatrics, 123, 1464–1471. 10.1542/peds.2008-0854 19482755 PMC2911576

[x24748307-20190625-01-bibr11] National Center for Health Statistics. (2016). Health, United States, 2016: With chartbook on long-term trends in health. Retrieved from Centers for Disease Control and Prevention website: https://www.cdc.gov/nchs/data/hus/hus16.pdf

[x24748307-20190625-01-bibr12] Nguyen T. H. Park H. Han H. R. Chan K. S. Paasche-Orlow M. K. Haun J. Kim M. T. (2015). State of the science of health literacy measures: Validity implications for minority populations. Patient Education and Counseling, 98, 1492–1512. 10.1016/j.pec.2015.07.013 PMC4732928 26275841

[x24748307-20190625-01-bibr13] Osborn C. Y. Wallston K. A. Shpigel A. Cavanaugh K. Kripalani S. Rothman R. L. (2013). Development and validation of the general health numeracy test (GHNT). Patient Education and Counseling, 91, 350–356. 10.1016/j.pec.2013.01.001 23433635 PMC3644342

[x24748307-20190625-01-bibr14] Peters E. Bjalkebring P. (2015). Multiple numeric competencies: When a number is not just a number. Journal of Personality and Social Psychology, 108, 802–822. 10.1037/pspp0000019 25285966

[x24748307-20190625-01-bibr15] Sauceda J. A. Loya A. M. Sias J. J. Taylor T. Wiebe J. S. Rivera J. O. (2012). Medication literacy in Spanish and English: Psychometric evaluation of a new assessment tool. Journal of the American Pharmacists Association, 52, e231–e240. 10.1331/JAPhA.2012.11264 23229985

[x24748307-20190625-01-bibr16] Stilley C. S. Terhorst L. Flynn W. B. Fiore R. M. Stimer E. D. (2014). Medication health literacy measure: Development and psychometric properties. Journal of Nursing Measurement, 22, 213–222. 10.1891/1061-3749.22.2.213 25255674 PMC4580338

[x24748307-20190625-01-bibr17] U.S. Census Bureau. (2014). Educational attainment in the United States: 2014. Retrieved from https://www.census.gov/data/tables/2014/demo/educational-attainment/cps-detailed-tables.html

[x24748307-20190625-01-bibr18] Wolf M. S. King J. Jacobson K. Di Francesco L. Bailey S. C. Mullen R. Parker R. M. (2012). Risk of unintentional overdose with non-prescription acetaminophen products. Journal of General Internal Medicine, 27, 1587–1593. 10.1007/s11606-012-2096-3 22638604 PMC3509295

[x24748307-20190625-01-bibr19] Yin H.S. Johnson M. S. Mendelsohn A.L. Abrams M. A. Sanders L. M. Dreyer B. P. (2009). The health literacy of parents in the United States: A nationally representative study. Pediatrics, 124, S289–S298. 10.1542/peds.2009-1162E 19861483

[x24748307-20190625-01-bibr20] Yin H. S. Parker R. M. Sanders L. M. Dreyer B. P. Mendelsohn A. L. Bailey S. Wolf M. S. (2016). Liquid medication errors and dosing tools: A randomized controlled experiment. Pediatrics, 138, e20160357. 10.1542/peds.2016-0357 27621414 PMC5051204

[x24748307-20190625-01-bibr21] Zong J. Batalova J., (2015). The limited English proficient population in the United States. Retrieved from Migration Information Source website: https://www.migrationpolicy.org/article/limited-english-proficient-population-united-states.

